# Deficiency of Brummer Impaires Lipid Mobilization and JH-Mediated Vitellogenesis in the Brown Planthopper, *Nilaparvata lugens*

**DOI:** 10.3389/fphys.2018.01535

**Published:** 2018-10-30

**Authors:** Kai Lu, Jinming Zhou, Xia Chen, Wenru Li, Yue Li, Yibei Cheng, Jing Yan, Keke You, Zhineng Yuan, Qiang Zhou

**Affiliations:** ^1^State Key Laboratory of Biocontrol, School of Life Sciences, Sun Yat-sen University, Guangzhou, China; ^2^College of Life Sciences, Fujian Agriculture and Forestry University, Fuzhou, China

**Keywords:** brummer, juvenile hormone, vitellogenesis, fecundity, *Nilaparvata lugens*

## Abstract

Provisioning of sufficient lipids and vitellogenin to the oocytes is an indispensable process for fecundity of oviparous insects. Acute mobilization of lipid reserves in insects is controlled by the Brummer (Bmm), an orthologous of human adipose triglyceride lipase (ATGL). To investigate the functional roles of brummer-mediated lipolysis in the fecundity of the brown planthopper, *Nilaparvata lugens*, RNA interference (RNAi) analyses were performed with double-stranded RNA (dsRNA) against *NlBmm* in adult females. Knockdown of *NlBmm* expression resulted in obesity and blocked lipid mobilization in the fat body. In addition, *NlBmm* silencing led to retarded ovarian development with immature eggs and less ovarioles, decreased number of laid eggs, prolonged preoviposition period and egg duration. Furthermore, severe reductions of vitellogenin and its receptor abundance were observed upon *NlBmm* knockdown. The transcript levels of *NlJHE* (juvenile hormone esterase) which degrades JH were up-regulated, whereas the expression levels of JH receptors *NlMet* (Methoprene-tolerant) and *NlTai* (Taiman) and their downstream transcription factors *NlKr-h1* (Krüppel-homolog 1) and *NlBr* (Broad-Complex) were down-regulated after suppression of *NlBmm*. JH-deficient females exhibited impaired vitellogenin expression, whereas JH exposure stimulated vitellogenin biosynthesis. Moreover, JH topical application partially rescued the decrease in vitellogenin expression in the *NlBmm*-deficient females. These results demonstrate that brummer-mediated lipolytic system is essential for lipid mobilization and energy homeostasis during reproduction in *N. lugens*. In addition to the classical view of brummer as a direct lipase with lipolysis activity, we propose here that brummer-mediated lipolysis works through JH signaling pathway to activate vitellogenesis and oocyte maturation that in turn regulates female fecundity.

## Introduction

In oviparous insects, maturation of oocytes requires the accumulation of large amounts of proteins and lipids in the yolk bodies and lipid droplets (Roy et al., 2017). Currently, significant progress has been achieved to understand the process of yolk protein, vitellogenin uptake by developing oocytes via receptor-mediated endocytosis from biosynthesis to hormonal regulation of vitellogenin gene expression (Tufail and Takeda, 2008, 2009; Belles and Piulachs, 2015). However, our knowledge on how lipids are accumulated in insect oocytes are still limited. Lipids, mostly triacylglycerol (TAG) stored in the oocytes are the main nutrient supply to provide energy for the development of embryo, to provide the precursors for the synthesis of cell membranes and to mediate cell signaling (Handela, 1993; Ziegler and Van Antwerpen, 2006; Santos et al., 2011). Although the enzymes accounting for TAG biosynthesis are active in insect oocytes, their ability to synthesize fatty acids (FAs) de novo is very limited (Van Hoof et al., 2005). Oocytes synthesize only small amounts of lipids and most lipids present in the eggs are transported from circulating lipoproteins in hemolymph (Parra-Peralbo and Culi, 2011). The main lipid carriers in the hemolymph are the lipophorin and vitellogenin, and most of the lipids are transferred via a lipophorin-mediated pathway (Canavoso and Wells, 2001). It has been demonstrated that the site of lipophorin and vitellogenin synthesis is the fat body, an insect organ analogous to vertebrate liver and adipose tissue and functions as a major organ for energy storage and metabolism (Arrese and Soulages, 2010).

The insect fat body stores surplus nutrient as lipids and glycogen, which is mobilized to meet the energy demand during vitellogenesis, embryogenesis, prolonged flights, and starvation stress (Canavoso et al., 2004; Liu et al., 2009; Wang et al., 2010). A balance between lipogenesis and lipolysis is key to match the acute energy demand during periods of excessive energy supply. In mammal, adipose triglyceride lipases (ATGLs) play critical roles in TAG mobilization, and ATGL mutation results in the accumulation of TAG and FAs in plasma (Zechner et al., 2007). In *Japanese quail*, ATGL knockdown shifts the balance toward a net accumulation of TAG in fat tissues and reduces number of eggs (Chen et al., 2016). At least two kinds of lipases have been documented to play important roles in the maintenance of insect lipid homeostasis by regulating lipolysis in the fat body. Both brummer lipase and adipokinetic hormone (AKH), a neurohormone with similar structural and functional characteristics of mammalian glucagon are required for the basal lipolytic activity of fat body (Grönke et al., 2005; Géminard et al., 2006). *Brummer*, the insect homolog of mammalian ATGL was first identified in the fruit fly, *Drosophila melanogaster* (Grönke et al., 2005) and the silkworm, *Bombyx mori* (Wang et al., 2010). In *Drosophila, brummer* mutant flies are obese with impaired lipid mobilization, whereas overexpression of *brummer* depletes lipid storage and results in lean flies (Grönke et al., 2007). Moreover, brummer is important for lipolysis in the fat body during insect starvation. Activated brummer interacts with perilipin at the lipid droplet membrane to eventually mobilize TAG is important for energy supply during starvation in *Drosophila* (Grönke et al., 2005, 2007). Silencing of *brummer* results in elevated levels of lipids at death after starvation in the tsetse fly, *Glossina morsitans* (Attardo et al., 2012). Lipolysis also operates to ensure efficient lipid and nutrient utilization during insect development and reproduction. In the cricket, *Gryllus bimaculatus* and the nematode, *Caenorhabditis elegans*, AKH injections lead to a decrease in the amount of fat body lipid stores and fewer terminal oocytes (Lorenz, 2003; Lorenz and Gäde, 2009). Silencing of *AKH* and its receptor delays egg maturation and reduces fecundity in *C. elegans* (Lindemans et al., 2009). In *Drosophila*, deficiency of brummer causes complete embryonic lethality (Grönke et al., 2005). *Brummer* knockdown females appear incapable of mobilizing lipid reserves during pregnancy leading to retarded oocyte development, reduced milk production, failed embryogenesis, and delayed larval development in *G*. *morsitans*, indicating that brummer-mediated lipolysis is involved in the regulation of tsetse reproduction (Attardo et al., 2012). Although the deficiency of brummer affects the fertility and body lipid metabolism of the insects, the detailed mechanism about how brummer regulates fecundity remains unknown.

In insects, female reproductive processes, from oogenesis to egg maturation are tightly controlled by endocrine hormones (Jindra et al., 2013; Belles and Piulachs, 2015). Juvenile hormone (JH), a sesquiterpenoid hormone synthesized and secreted by the corpora allata (CA), regulates larva metamorphosis and participates in adult female reproductive maturation (Riddiford, 2008; Jindra et al., 2015). It has been documented that JH stimulates the storage of lipids in the oocytes by promoting the biosynthesis and secretion of the lipid carrier proteins, mainly vitellogenin from the fat body and by facilitating the uptake of vitellogenin (Davey, 2007; Lu et al., 2015). Large amounts of vitellogenin are synthesized in the fat body and secreted to the hemolymph after protein posttranslational modification during vitellogenesis (Tufail and Takeda, 2008). Then, vitellogenin is incorporated into developing oocytes via receptor-mediated endocytosis, deposited in yolk bodies and used for the development of embryo (Tufail and Takeda, 2009). Meanwhile, vitellogenin is a phospholipoglycoprotein which contributes to the final deposits in the mature eggs, and 5% of lipids in the oocytes originated from endocytosed vitellogenin, whereas lipophorin accounting for nearly 95% of lipid storage in mature oocytes (Canavoso and Wells, 2001; Ziegler and Van Antwerpen, 2006). In mosquito, JH prevents follicular resorption and stimulates female fecundity through promoting the accumulation of ovarian lipids (Clifton et al., 2014). In addition, JH seems to promote the expression of lipophorin receptor (LpR) at the translational level in *Blattella germanica* (Maria-Dolors et al., 2007). Lipids accumulated in eggs must be efficiently broken down by lipases to provide energy supply and metabolic intermediates during embryogenesis. In *D. melanogaster*, it was reported that JH controls the expression of genes involved in lipogenesis and lipolysis to support increased lipid metabolism and enhanced reproductive output (Reiff et al., 2015). In the brown planthopper, *Nilaparvata lugens*, it was proposed that brummer is highly expressed in ovaries and may also be involved in lipid mobilization during oogenesis (Zhou et al., 2018a). Although these results provide evidence that lipases and JH are required for the lipid accumulation by developing oocytes, little is known about endocrine regulation and the physiological functioning of lipolysis during this period. A thorough understanding of the relationship between brummer-dependent lipid mobilization and their functions in JH-mediated female fecundity would be of great value in promoting future research in insect reproduction.

In the present study, we aim to reveal the functional roles of brummer in lipid mobilization and female fecundity in *N. lugens*, one of the most important rice pests. To address this issue, we evaluated the parameters of lipid metabolism and female reproductive biology after knocking down brummer. In addition, the involvement of brummer in the regulation of JH-mediated vitellogenesis was also investigated. The obtained data show that brummer is indispensable for lipid mobilization and reproductive maturation in females, and that brummer controls vitellogenesis by regulating the expression of JH-related pathway genes in *N. lugens*.

## Materials and methods

### Insect rearing

*N. lugens* was originally collected from the experimental rice fields in South China Agricultural University in August 2014. Insects were reared on Zengcheng rice seedlings at 26 ± 1°C, 70–80 % relative humidity and a 14/10 h (light/dark) photoperiod in a climatic chamber. Newly emerged females (within 24 h) were collected and kept isolated until they were used for experiments.

### RNA extraction, cDNA synthesis and real-time quantitative PCR (qPCR)

Total RNAs were extracted from insect whole bodies or tissues using Trizol reagent (Invitrogen, California, USA). RNA concentration was measured using a Nanodrop 2000C spectrophotometer (Thermo Fisher Scientific, West Palm Beach, FL, USA). The total RNA was treated with DNase I (Promega, Madision, WI, USA) to remove contaminating genomic DNA. First-strand cDNA was synthesized from 5 μg of total RNA using the GoScript Reverse System (Promega, Madision, WI, USA) with random primers and oligo (dT) in a total reaction volume of 10 μL according to the manufacturer's instructions.

Gene-specific primers used for RT-PCR and qPCR were designed by Primer 6 software and presented in Table 1. For RT-PCR, a 498 bp fragment of *NlBmm* was amplified with GoTaq Master Mix (Promega, Madison, WI, USA) using the following cycling conditions: 2 min at 95°C, followed by 30 cycles of 30 s at 95°C, 30 s at 60°C and 45 s at 72°C and a final extension of 5 min at 72°C. PCR products were separated with 1.5% agarose gel electrophoresis and visualized by ethidium bromide staining. The *N. lugens* β*-actin* (EU179850) was used as the internal control. All the qPCR experiments were performed in 10 μL reaction mixtures containing 5 μL of GoTaq qPCR Master Mix (Promega, Madison, WI, USA), 0.4 μL of each primer (10 μM), 3.2 μL of nuclease-free water and 1 μL of cDNA template. qPCR was performed in 384-well plates using a Light Cycler 480 instrument (Roche Diagnostics, Basel, Switzerland) under the following reaction conditions: 1 cycle of 95°C for 30 s, followed by 40 cycles of 95°C for 5 s, 60°C for 15 s, and 72°C for 20 s. At the end of each qPCR, a melting curve analysis (65–95°C) was carried out to check the amplification specificity. All qPCR experiments were performed in triplicate, and at least two technical replicates were performed in each biological sample. The relative expression levels were normalized against β*-actin* (EU179850) and *GAPDH* (KU058667) (Lu et al., 2018) and calculated using 2^−ΔΔ*CT*^ method (Livak and Schmittgen, 2001).

**Table 1:** Primers used in this study.

**Primers**	**Primer sequence**
***For RT-PCR***	
Bmm-F	5′-TGATTCCGCCCAAGTTCCAC-3′
Bmm-R	5′-TCCAGTTGATGACGCCCTTG-3′
Actin-F	5′-GCCGCGATCTGACCGACTAC-3′
Actin-R	5′-TGAGGGAGCGAGGGAAGTGA-3′
***For qRT-PCR***	
qBmm-F	5′-ACTGTGAGTCCGTTCTGCG-3′
qBmm-R	5′-AACCGATGTATGTTCTGCTT-3′
qJHE-F	5′-GTGGGCAGACCTACCGCAGGG-3′
qJHE-R	5′-GCGAGAGCCGCGTGCATTGC-3′
qJHAMT-F	5′-GAACCTGCAGGCCAAACACA-3′
qJHAMT-R	5′-ACCACTCGGTTGGGCTGAAT-3′
qMet-F	5′-AGTGGCAGCGAGCGATGATT-3′
qMet-R	5′-TGAGGCGCAGCAAAAAGGAG-3′
qTai-F	5′-ATGATCCCAACCACTTCAGC-3′
qTai-R	5′-TTCCACTCACACTACCACCA-3′
qKr-h1-F	5′-TGATGAGGCACACGATGACT-3′
qKr-h1-R	5′-ATGGAAGGCCACATCAAGAG-3′
qBr-F	5′-CCAGGCAAACAACCCAATC-3′
qBr-R	5′-CTACACTGCCCCTCTTCACG-3′
qVg-F	5′-TTCCGTTTGCAGCCACCTATG-3′
qVg-R	5′-CTGCTGCTGCTGCTTCTGTCA-3′
qVgR-F	5′-AGGCAGCCACACAGATAACCGC-3′
qVgR-R	5′-AGCCGCTCGCTCCAGAACATT-3′
qβ-actin-F	5′-CCCTCGCTCCCTCAACAATG-3′
qβ-actin-R	5′-TGGATGGACCAGACTCGTCGT-3′
qGAPDH-F	5′-CCGTCAGACTGGGCAAGGAC-3′
qGAPDH-R	5′-GCGCGTCGAAGATGGAAGAG-3′
***For dsRNA Synthesis***	
Bmm-Fi	5′-ggatcctaatacgactcactatagggCTCTCGTTTGCGGGAT GTGGATTCT-3′
Bmm-Ri	5′-ggatcctaatacgactcactatagggCCTCAGGGCATCGTCA AAACC-3′
GFP-Fi	5′-ggatcctaatacgactcactatagggAAGGGCGAGGAGCTG TTCACCG-3′
GFP-Ri	5′-ggatcctaatacgactcactatagggCAGCAGGACCATGTG ATCGCGC-3′

*F, forward primer; R, reverse primer. Lowercase letters indicate the T7 promoter sequences*.

### Triacylglycerol (TAG) detection and nile red staining

TAG and glyceride contents were measured as described previously (Zhou et al., 2018a). Briefly, 6-8 female whole bodies or fat bodies from 15 females were collected and homogenized in PBS containing 0.5% Tween 20 (PBST), then centrifuged and the supernatants were collected. TAG was quantified using Triglyceride Determination Kit and glyceride contents were measured with Free Glycerol Reagent (Sigma-Aldrich, St Louis, MO, USA) according to the manufacturer's instructions. Nile red staining experiments were performed as described previously (Zhou et al., 2018b). Briefly, fat bodies from dsRNA-injected females on the 6th day after injection were carefully dissected out in precooled phosphate buffered saline (PBS) buffer, and then fixed by submerging in 4% paraformaldehyde for 2 h at room temperature. After washing the fat body three times (3 × 5 min) with PBS, the tissues were stained with Nile red solution [1 μL of Nile red stock solution (1 mg/ml) in 100 μL of PBS] for 10 min at room temperature. After washing for 3 × 5 min with PBS again, the fat body was mounted in 75% glycerol and imaged using a Ti-S inverted fluorescence microscope (Nikon, Tokyo, Japan).

### RNA interference

RNA interference (RNAi) was performed as described previously (Zhou et al., 2018a). Briefly, dsRNA was synthesized by T7 RiboMAX™ Express RNAi System (Promega Corporation, Madison, WI, USA) using primers with T7 promoter sequences for *NlBmm* specific gene amplification (Table 1). The *GFP* gene (ACY56286) was used as a control dsRNA for the check of RNAi specificity (Chen et al., 2010). The quality and concentration of dsRNAs were determined with Nanodrop2000C (Thermo Fisher Scientific, West Palm Beach, FL, USA), and the size of the dsRNAs was measured by 1.5 % agarose gel electrophoresis. Newly emerged females were injected with 400 ng dsRNA using a Nanoject II microinjection device (Drummond Scientific, Broomall, PA, USA). Females injected with dsRNA were reared on fresh rice seedlings under the conditions described above. Knockdown efficiency was confirmed in each experiment using qPCR on the 3rd and 6th day after dsRNA injection. Three independent biological replicates were performed in each treatment.

### Protein isolation and western blot

Whole bodies of *N. lugens* were homogenized in lysis buffer (8 M urea, 4% CHAPS, 40 mM Tris-HCL, 5 mM EDTA, 1 mM PMSF, 10 mM DTT, and 0.2 mM protease inhibitor, pH 8.0) using a TGrinder tissue homogenizer (Tiangen, Beijing, China). The homogenates were centrifuged at 12,000 ×g for 1 h at 4°C and the protein supernatant was collected. Protein concentrations were measured using Bradford assay kit (Tiangen, Beijing, China). Thirty micrograms of total protein were loaded in each lane of a 10% SDS-PAGE and then transferred to 0.45 μm polyvinylidene difluoride membrane (PVDF) membrane (Millipore, Billerica, MA, USA). The membranes were blocked with 5% (w/v) milk in Tris-buffered saline (TBS) at room temperature for 2 h, then immunoblotted with *Nl*Vg primary antibody (1:5000), *Nl*VgR primary antibody (1:1000), or β-actin antibody (1:2000) at 4°C overnight. After washing with PBST for three times, the membrane was incubated with goat anti-rabbit immunoglobulin G horseradish peroxidase-linked secondary antibody (Sigma-Aldrich, St Louis, MO, USA) at room temperature for 1 h. The immunoreactivity was visualized with enhanced chemiluminescence system Super Signal West Pico (Pierce, Rockfod, IL) and photographed by the GBOX-Chemi XT4 imaging system (Syngene, Cambridge, UK) as described previously (Lu et al., 2015).

### Fecundity assay and ovary dissection

Biological performance parameters were measured as previously reported with moderate modification (Ge et al., 2015). For fecundity analysis, each female injected with dsRNA was crossed to two healthy males and then transferred onto fresh rice seedlings for oviposition under the conditions described above. The males were removed 6 days later, and the rice seedlings were dissected under the microscope every day to count the number of laid eggs. The preoviposition period is the time from adult emergence to the onset of egg-laying. For egg duration analysis, one female treated with dsRNA together with two males were put onto rice seedlings to produce offspring. The females were removed 10 days later, and the number of hatched offspring was counted daily. The egg duration is the time from the onset of egg-laying to the adult emergence. Twelve females were analyzed per group and three independent replicates were carried out for each target gene. Ovaries were dissected in the precooled PBS buffer from 10 to 12 randomly selected living dsRNA-injected females. The adherent tissues of the ovary were carefully removed with forceps as thoroughly as possible and observed on a SMZ18 stereomicroscope (Nikon, Tokyo, Japan).

### Juvenile hormone experiments

Two-day-old females were anesthetized with carbon dioxide and topically applied with 250 ng JH III (Sigma, St. Louis, MO, USA) dissolved in 50 nL acetone using a Nanoject II microinjection device (Drummond Scientific, Broomall, PA, USA). Control females were treated with the same volume of acetone. Then the treated females were kept on rice seedlings under the conditions described above. The mRNA levels of *NlVg* was calculated by qPCR and *Nl*Vg protein abundance was detected using western blot 24 h later. For the analysis of effects of JH III on the *NlVg* expression influenced by *NlBmm*, newly emerged females were injected with dsRNA (dsBmm or dsGFP). Three days after the dsRNA injection, the treated females were applied with JH III and *Nl*Vg expression levels were determined 24 h later as described above.

### Statistical analysis

Values were expressed as means ± SE based on three independent biological replicates. Data were analyzed by Student's *t*-test for the comparison of two different conditions. Differences were considered significant at *p* < 0.05 (^*^) and *p* < 0.01 (^**^). All statistical analyses were performed using SPSS 18.0 software (SPSS Inc., Chicago, IL, USA).

## Results

### Knockdown of *NlBmm* by RNAi

**Figure 1:**
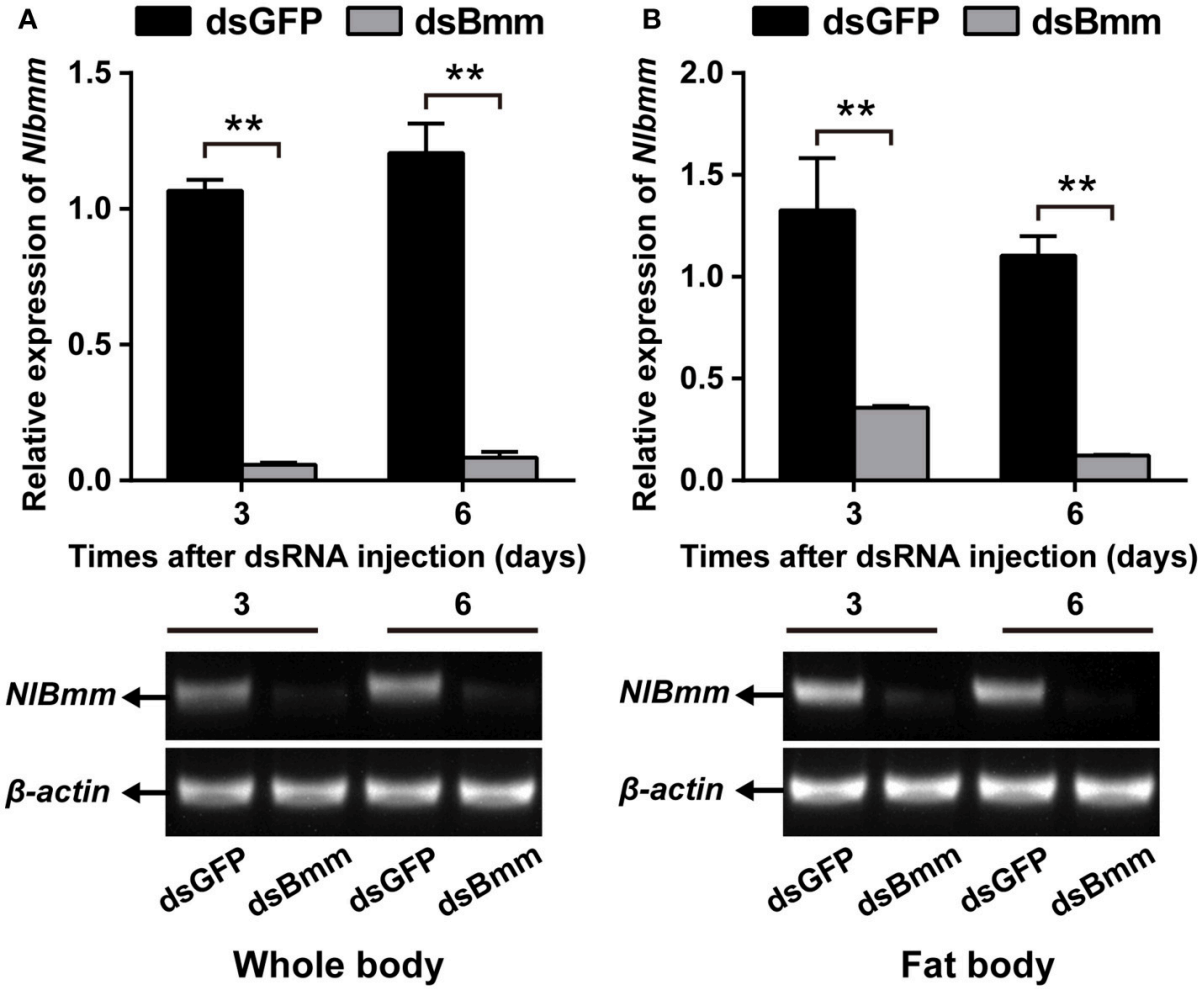
Double-stranded RNA (dsRNA)-mediated silencing of *NlBmm* transcript in *N. lugens*. The knockdown efficiency of *NlBmm* in the whole body **(A)** and fat body **(B)** at different days after dsRNA injection. Data are presented as means ± SE based on three independent experiments. Bars annotated with asterisks indicate significant differences between the control and treated groups (*P < 0.05; **P < 0.01) by Student's *t*-test.

To investigate the role of *NlBmm* in lipid metabolism and female fecundity, we performed a dsRNA-mediated knockdown of *NlBmm* and evaluated the impact on TAG content in the fat body. The RNAi efficiency was confirmed by RT-PCR and qPCR by using RNA extracted from the whole bodies or the dissected fat bodies. The effects of RNAi in the whole bodies were determined with a reduction in *NlBmm* expression of more than 90% on day 3 and 6 after dsRNA injection (Figure 1A). When compared to dsGFP-injected controls, *NlBmm* expression level in the fat bodies of *NlBmm*-deficient females was 73.05 and 88.84% lower on the 3rd and 6th day after dsRNA injection, respectively (Figure 1B).

### *NlBmm* knockdown reduces *N. lugens* fecundity

**Figure 2:**
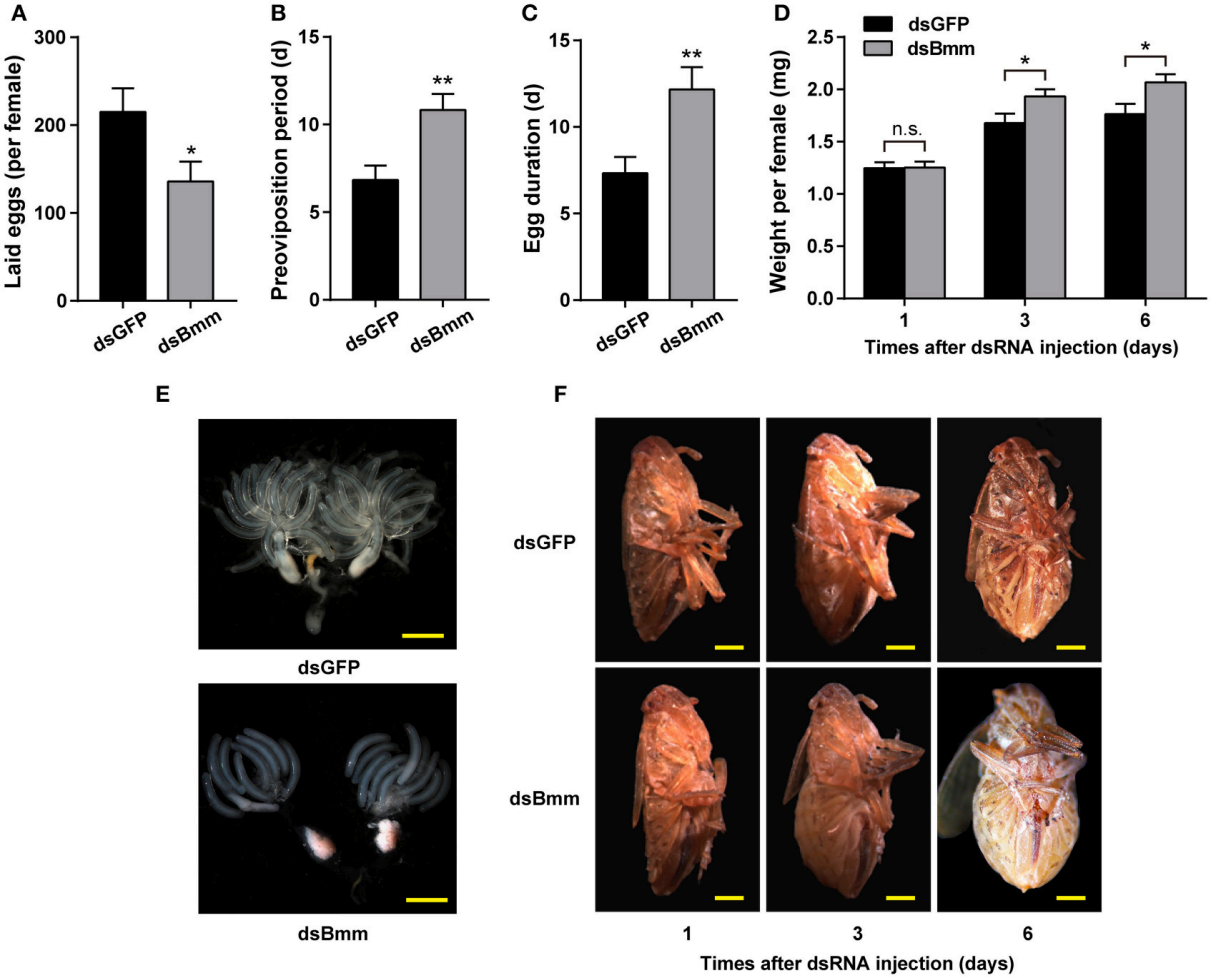
Reduced fecundity and severe obesity in *NlBmm*-knockdown females. Newly emerged females were injected with dsRNA for *NlBmm* or *GFP* (control) genes and reared on fresh rice seedlings under normal conditions. The oviposition and hatching were monitored. Number of eggs laid per female **(A)**, preoviposition periods **(B)** and egg duration **(C)** were determined using a dissection microscopy (*n* = 12). **(D)** The average body weight of silenced and control females was determined after dsRNA injection (*n* = 18–19). Representative images from ovaries **(E)** and females **(F)**. Scale bar, 500 μm. Data are means ± SE of three independent biological replicates and asterisks indicate significant differences between the control and treated groups (*P < 0.05; **P < 0.01) by Student's *t*-test.

To investigate whether the knockdown of *NlBmm* can affect female fecundity, we analyzed the egg-laying, preoviposition period and egg duration with dsBmm- and dsGFP-treatment. The number of total eggs laid by dsBmm-injected females was 136, which represents a 36.77% reduction compared with that in controls (Figure 2A). The preoviposition period of the *NlBmm*-RNAi females was significantly prolonged by 4 days compared with the dsGFP-injected controls (Figure 2B). In addition, the egg duration in *NlBmm*-deficient females was also prolonged significantly by 4.83 days (Figure 2C). *NlBmm* knockdown resulted in retarded ovarian development with immature eggs and less ovarioles, while dsGFP-injected control females showed fully-developed ovaries and possessed plentiful mature eggs (Figure 2E). Silencing *NlBmm* showed no significant effects on the initial weight (1 day after dsRNA injection). However, compared to dsGFP-injected controls, females lacking *Nl*Bmm lipase activity develop normally but show progressive obesity accumulating 15.05% (immature adults, 3 day after dsRNA injection) and 17.21% (mature adults, 6 days after dsRNA injection) more weight, consistent with the females' inability to mobilize storage fat (Figures 2D,F).

### *NlBmm* knockdown impairs lipid mobilization

**Figure 3:**
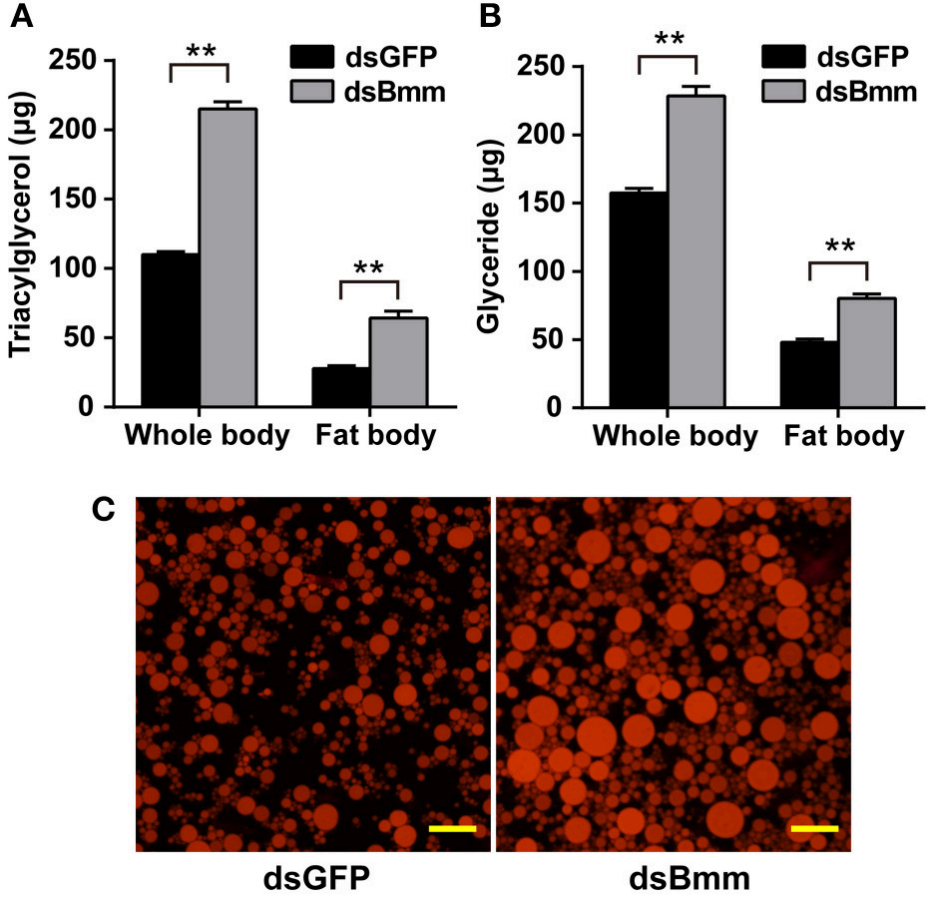
*NlBmm* knockdown increases TAG and glyceride contents in the whole body **(A)** and fat body **(B)**. Newly emerged females were injected with dsRNA for *NlBmm* or *GFP* (control) genes. The insects were fed on fresh rice seedlings after dsRNA injection and the fat body was dissected 6 days later. Whole bodies and fat bodies were homogenized in PBS solution and TAG and glyceride were determined. Data are means ± SE of three independent determinations and asterisks indicate significant differences between the control and treated groups (*P < 0.05; **P < 0.01) by Student's *t*-test. **(C)** Nile-red staining of the lipid storage droplets in the fat body of the dsRNA-injected females. Scale bar, 20 μm.

*NlBmm*-deficient females accumulated more TAG (1.96-fold) compared with the dsGFP-injected control females. The fat body is the main organ for lipid storage and metabolism in insect. To better understand the role of *NlBmm* in lipid mobilization, we also measured TAG content in the fat bodies of females after *NlBmm* knockdown. For *NlBmm*-silenced females, the fat body TAG level increased (2.31-fold) on day 3 after dsRNA injection compared with that in dsGFP-injected controls (Figure 3A). Similar results were also observed in glyceride content after *NlBmm* knockdown (Figure 3B). Nile red staining was performed to visualize lipid droplets in the fat body to measure the stored lipid contents. Our results showed that fat bodies from *NlBmm*-deficient females accumulated larger and higher density of lipid droplets compared with that in dsGFP-injected controls (Figure 3C).

### *NlBmm* knockdown suppresses vitellogenesis

**Figure 4:**
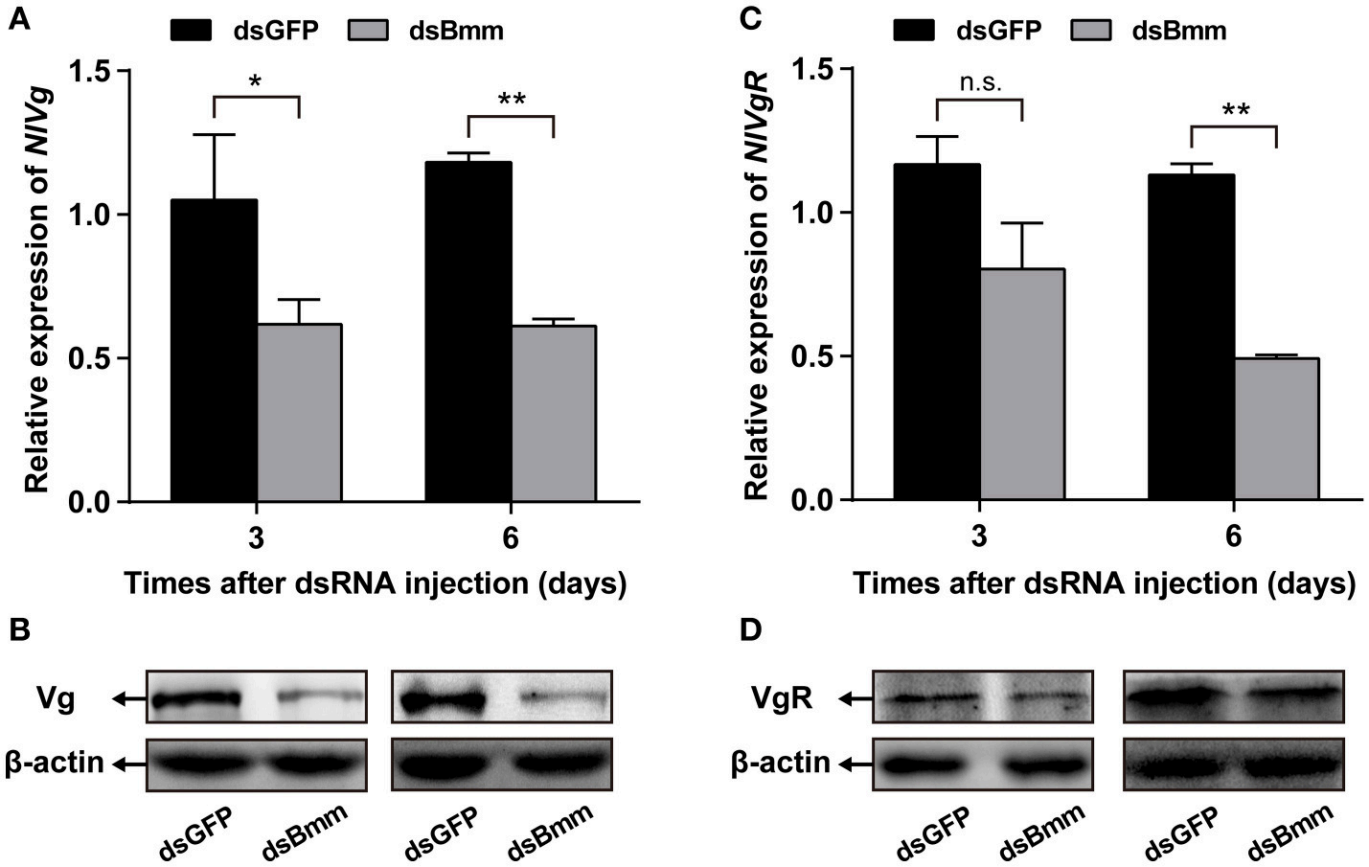
*NlBmm* knockdown reduces Vg and VgR expression. Newly emerged females were injected with dsRNA for *NlBmm* or *GFP* (control) genes. Transcript levels and protein abundance of *Nl*Vg **(A,B)** and *Nl*VgR **(C,D)** in whole bodies were determined by qRT-PCR and western blot on the 3rd and 6th day after *NlBmm* knockdown. *N. lugens* β-actin expression levels were set as the internal control. Data are means ± SE of three independent determinations and asterisks indicate significant differences between the control and treated groups (*P < 0.05; **P < 0.01) by Student's *t*-test.

Compared to control females, *NlVg* expression level in the *NlBmm*-deficient females was 50.44 and 48.22% lower on the 3rd and 6th day after dsRNA injection, respectively (Figure 4A). Significant reductions in *Nl*Vg protein levels were also detected in the *NlBmm*-deficient females by western blot (Figure 4B). To ascertain the impact of *NlBmm* knockdown on Vg endocytosis into oocytes, we also investigated the expression of Vg receptor (VgR) of dsRNA treated females. The transcript abundance of *NlVgR* was 56.52% lower than in control insects on the 6th day after dsRNA injection (Figure 4C). Moreover, *NlBmm* knockdown resulted in a significant reduction in *Nl*VgR protein abundance (Figure 4D).

### *NlBmm* knockdown affects the expression of JH-related genes

**Figure 5:**
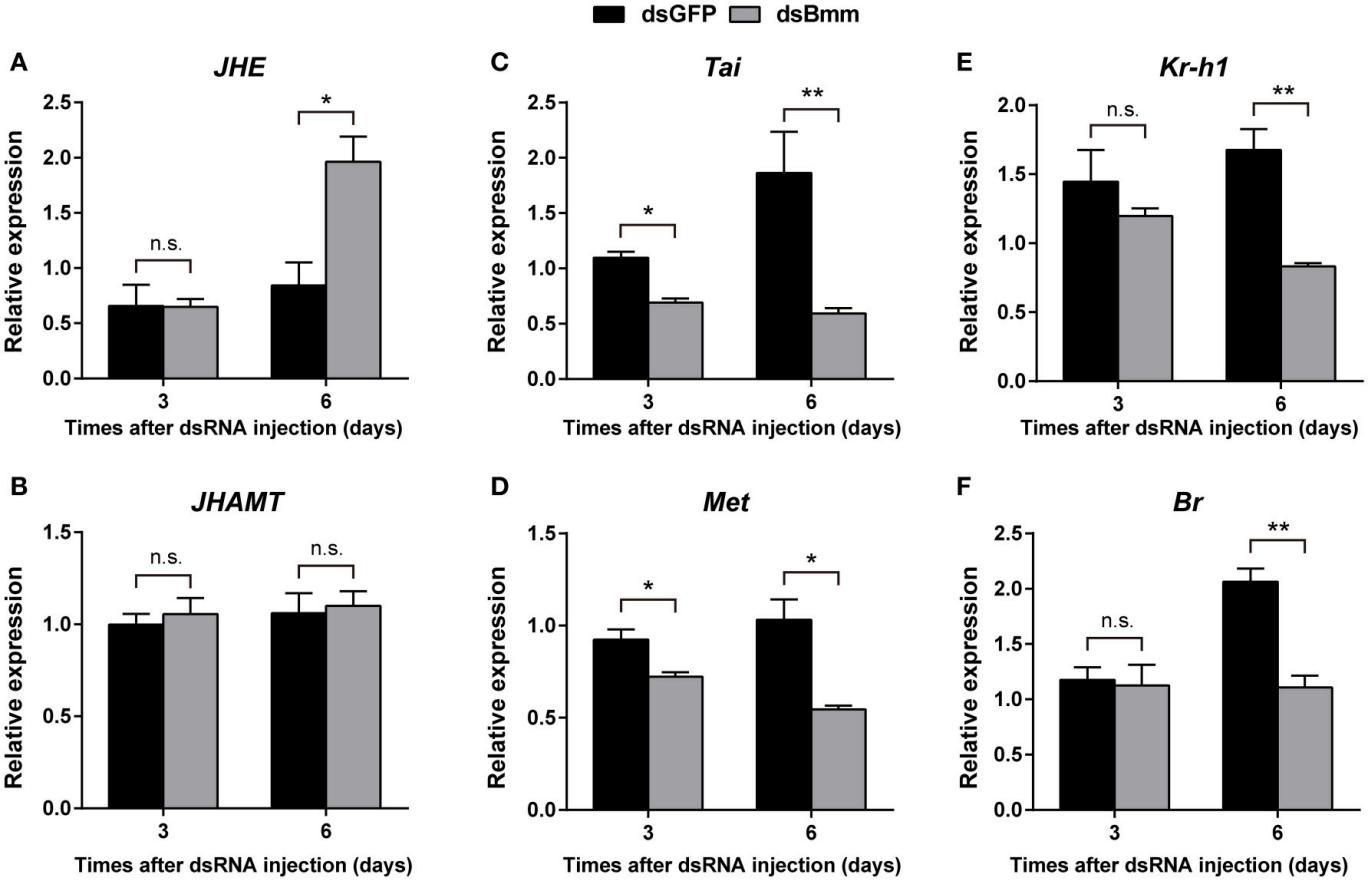
Effect of *NlBmm* knockdown on the expression of JH pathway-related genes. Newly emerged females were injected with dsRNA for *NlBmm* or *GFP* (control) genes. Transcript levels of JH pathway-related genes in whole bodies were determined by qRT-PCR on the 6th day after *NlBmm* knockdown. Data are means ± SE of three independent experiments and asterisks indicate significant differences between the control and treated groups (*P < 0.05; **P < 0.01) by Student's *t*-test. **(A)** JHE, juvenile hormone esterase; **(B)** JHAMT, juvenile hormone acid methyltransferase; **(C)** Tai, Taiman; **(D)** Met, Methoprene-tolerant; **(E)** Kr-h1, Krüppel-homolog 1; **(F)** Br, Broad-Complex.

To determine whether JH pathway was affected by the deficiency of *NlBmm*, we measured the expression levels of several JH pathway-related genes after *NlBmm* silencing. In the *NlBmm*-deficient females, the transcript abundance of *NlJHE* which degrades JH was 2.33-fold higher than in dsGFP-injected controls on day 6 after dsRNA injection. No significant variations in *NlJHAMT* mRNA levels were detected after *NlBmm* knockdown. Compared to control females, the expression levels of two JH receptor genes (*NlTai* and *NlMet*) were decreased significantly on the 3rd and 6th day after dsRNA injection. The expression levels of *NlKr-h1* and *NlBr*, two important down-stream transcription factors of JH signaling pathway were 50.32 and 46.29% lower in *NlBmm*-deficient females than in controls on the 6th day after dsRNA injection, respectively (Figure 5).

### JH rescue for *NlBmm*-deficient females

**Figure 6:**
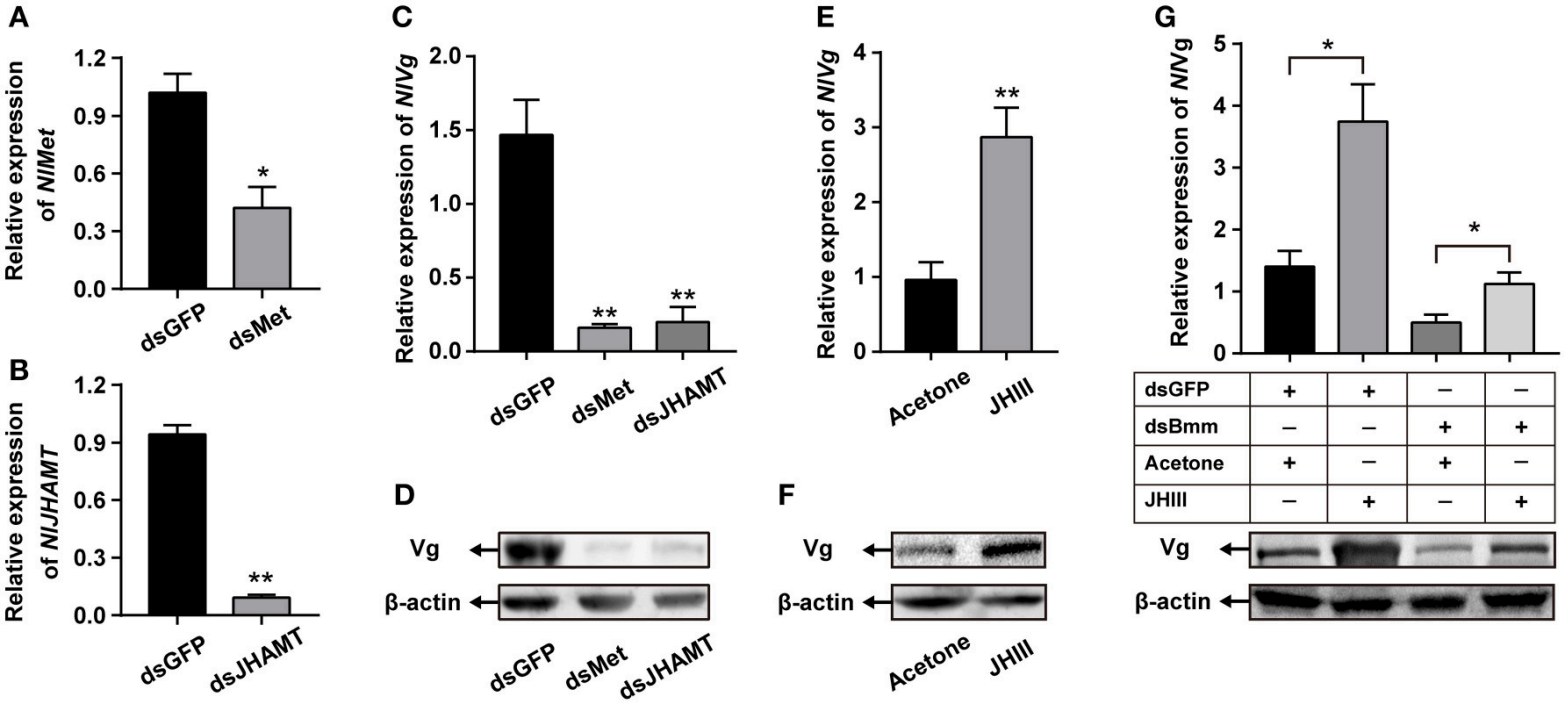
Application of JH partially restored Vg synthesis after *NlBmm* knockdown. Newly emerged females were injected with dsRNA for *NlMet, NlJHAMT* or *GFP* (control) genes. The RNAi efficiencies of *NlMet* **(A)** and *NlJHAMT* **(B)** were measured on the 3rd day after dsRNA injection. *NlVg* transcript levels **(C)** and *Nl*Vg protein abundance **(D)** were determined on the 3rd day after dsRNA injection. Each two-day-old female was treated with 250 ng JH III (dissolved in acetone) or the same volume acetone (50 nL, control), and *NlVg* transcript levels **(E)** and *Nl*Vg protein abundance **(F)** were determined 24 h later. **(G)** *Nl*Vg protein abundance of dsBmm-injected females further topically applied with JH III. Newly emerged females were injected with dsRNA for *NlBmm* or *GFP* (control) genes and reared for two days. After that, the females were topically treated with JH III or acetone, and *Nl*Vg protein levels were determined by western blot at 24 h. *N. lugens* β-actin expression levels were set as the internal control. Data are means ± SE of three independent determinations and asterisks indicate significant differences between the control and treated groups (*P < 0.05; **P < 0.01) by Student's *t*-test.

We performed a JH rescue with *NlBmm*-deficient females in order to determine whether JH is the regulator of *NlBmm*-dependent vitellogenesis. In order to reveal the roles of JH-related gens in regulating vitellogenin biosynthesis in *N. lugens*, we first knocked down the expression of *NlMet* and *NlJHAMT* and then evaluated the impacts on Vg expression. The RNAi efficiencies were measured by qPCR with a reduction on *NlMet* expression of 58.73% (Figure 6A) and a reduction on *NlJHAMT* expression of 90.2% (Figure 6B) after dsRNA injection when compared with dsGFP-injected control. *NlMet* or *NlJHAMT* knockdown severely reduced *NlVg* mRNA expression levels (Figure 6C) and *Nl*Vg protein abundance (Figure 6D). On the other hand, JH III application significantly elevated the mRNA (Figure 6E) and protein abundance of *Nl*Vg (Figure 6F) at 24 h after treatment. JH III topical application partially rescued the decrease in *Nl*Vg expression in the *NlBmm*-deficient females (80.08%) compared with acetone-treated controls (35.4%) (Figure 6G).

## Discussion

In the present study, we found that the fecundity of *N. lugens* was significantly decreased after *brummer* knockdown. That insect reproduction is regulated by brummer-mediated lipolysis has been reported before. In the tsetse, *G. morsitans*, brummer plays an important role in the maintenance of hemolymph lipid levels and regulates milk production during tsetse pregnancy (Attardo et al., 2012). Our results demonstrate that both lipid mobilization and JH-mediated vitellogenesis influenced by brummer are involved in the positive control of female fecundity. To our knowledge, this is the first report of a functional role for the brummer-mediated vitellogenesis in insect reproduction.

Female reproduction, especially production of mature eggs is one of the most energy demanding events in the adult life of oviparous insects (Roy et al., 2017). The fat storage and energy mobilization are both function in egg development. In *A. aegypti*, the majority of stored lipids in the mature oocytes is TAG, and the ability to accumulate and mobilize lipid reserves is especially important for egg maturation (Ziegler, 1997; Ziegler and Ibrahim, 2001). Both the AKH/AKHR and brummer-mediated lipolytic systems are essential for lipid provision and nutrient transfer during reproduction. In the oriental fruit fly, *Bactrocera dorsalis*, silencing of *AKHR* resulted in the lowered lipolytic activity and delayed oocyte maturation, and the decreased fecundity may be attributed to the inability of utilizing lipid reserves of fat body to fuel oocyte maturation (Hou et al., 2017). In the tsetse fly, *G. morsitans*, the females with AKHR and brummer knockdown show incapable of mobilizing lipid reserves in fat body during pregnancy, slowered rate of oocyte development and failed embryogenesis, which indicates that the fecundity of females depends on lipid metabolism regulated by AKHR (Attardo et al., 2012). In *N. lugens*, silencing of brummer during vitellogenesis led to a rapid increase in lipid storages in fat body than that of in normal females (Figure 3). This supports the idea that reproductive disruption from brummer knockdown is due to the inability of female to mobilize lipid reserves in fat body required for oocyte maturation during vitellogenesis. Unfortunately, the functional relevance of this decreased lipid mobilization in females fecundity is still unknown.

On the other hand, the increased rate of lipolysis also negatively affect insect fecundity. That female reproduction is controlled by AKH signaling has been reported in a few insect species. In the locust, *Schistocerca gregaria* (Gokuldas et al., 1988), and the mosquito, *A. aegypti* (Ziegler, 1997), AKH exposure resulted in accelerated utilization of lipid reserves and reduced fecundity. Of interest is that AKH suppressed the synthesis of several specific proteins closely related to female reproduction (Carlisle and Loughton, 1979; Moshitzky and Applebaum, 1990). More recently, the role of AKH during insect vitellogenesis has been studied in several insect species. In the cricket, *G. bimaculatus* (Lorenz, 2003; Lorenz and Gäde, 2009) and the nematode, *C. elegans* (Lindemans et al., 2009), injections of AKH interfered with egg production by inhibiting the expression of vitellogenins and the accumulation of lipid stores. These results suggest that lipid homeostasis appears critical for insect fecundity as its disruption dramatically suppresses egg production and negatively affects vitellogenesis. In our previous study, knockdown of lipophorin receptor (LpR) caused retarded oocyte maturation and decreased fecundity (Lu et al., 2018). This phenotype is a probable result of reduced TAG accumulation and vitellogenin biosynthesis in the fat body. Here, we found that brummer plays an important functional role in *N. lugens* lipid mobilization, ovary development and fecundity. Our results indicated that females lacking brummer exhibit significantly reduced levels of vitellogenin and its receptor (Figure 4). Given the importance of vitellogenin biosynthesis and transportation required for oocyte maturation (Lu et al., 2015), it appears that the vitellogenesis mediated by brummer also functions in female fecundity. Taken together, these results clearly demonstrated that lipid homeostasis is essential for the vitellogenin biosynthesis in the fat body and for its incorporation by oocyte. Since the vitellogenesis is strongly linked with insect hormone JH (Roy et al., 2017), it is possible that brummer-based lipolysis possesses an important modulatory effect on JH signaling pathway during *N. lugens* vitellogenesis.

It is well documented that JH plays important roles in the control of larva development and adult female reproduction in insects (Riddiford et al., 2003). The homeostasis of JH titer is generally achieved by the combined effect of JH biosynthesis and degradation (Bellés et al., 2005). In order to evaluate the possible relationship between brummer-mediated lipolysis and JH-dependent vitellogenesis, the expressions of JH signaling pathway-related genes were determined after knockdown of brummer. We show that reducing brummer by RNAi in *N. lugens* resulted in the increased expression of *NlJHE*, an esterase responsible for JH degradation (Liu et al., 2008). In addition, the expressions of the JH receptors *NlMet* and *NlTai* (Lin et al., 2017) and their downstream targets *NlKr-h1* and *NlBr* (Jiang et al., 2017) were down-regulated after suppression of brummer (Figure 5). Moreover, JH topical application partially rescued the decrease in vitellogenin expression in the *NlBmm*-deficient females (Figure 6). These results suggest that brummer-lipolysis is especially important for vitellogenesis and this regulation may be mediated by JH. However, so far there is no clear indication which role that brummer may play in the regulation of JH-dependent vitellogenesis. The fat body is a specific important tissue which senses and integrates various nutritional and hormonal signals required for the regulation of vitellogenesis (Arrese and Soulages, 2010). Our previous studies have demonstrated that nutritional signaling pathway induces JH biosynthesis that in turn stimulates vitellogenin expression in the fat body and egg production in *N. lugens* (Lu et al., 2016a,b). These results promote us to speculate that brummer-mediated lipolysis works through nutritional signaling pathway to activate JH signaling that in turn regulates vitellogenesis and oocyte maturation. Total amount of lipids present in the fat body is a balance between lipogenesis and lipolysis (Grönke et al., 2005), and both pathways are essential for lipid homeostasis. Disruption of either lipogenesis or lipolysis could interfere with nutritional and physiological conditions of fat body and negatively affects JH-dependent vitellogenesis. Similarly, LpR-mediated lipid uptake by fat body is required to maintain the activity of nutritional signaling pathway and the elevated levels of vitellogenin biosynthesis (Lu et al., 2018). Maintenance of lipid levels of fat body in response to the demand by the developing oocyte during vitellogenesis also plays key roles in the regulation of nutritional and hormonal pathways.

In conclusion, our results provide a scientific link of lipolysis and fecundity to the function of brummer in nutrient-sensing and hormonal regulation mechanisms in the female adults of *N. lugens*. Since the lipid metabolism and vitellogenesis are crucial events for oocyte maturation and egg production, understanding the functional role of brummer could contribute to the elaboration of pest control strategies. Certainly, more studies are required to elucidate the direct function of brummer in the regulation of JH-dependent vitellogenesis.

## Author contributions

KL, JZ, and QZ designed the research and wrote the paper. XC, WL, YL, and YC designed and performed most of the experiments. JY, KY, and ZY performed western blot and analyzed the data. All authors read and approved the manuscript for publication.

### Conflict of interest statement

The authors declare that the research was conducted in the absence of any commercial or financial relationships that could be construed as a potential conflict of interest.
